# Acid Sphingomyelinase Inhibition Stabilizes Hepatic Ceramide Content and Improves Hepatic Biotransformation Capacity in a Murine Model of Polymicrobial Sepsis

**DOI:** 10.3390/ijms19103163

**Published:** 2018-10-15

**Authors:** Ha-Yeun Chung, C. Julius Witt, Jorge Hurtado-Oliveros, Jonathan Wickel, Markus H. Gräler, Amelie Lupp, Ralf A. Claus

**Affiliations:** 1 Center for Sepsis Control and Care, Jena University Hospital, 07747 Jena, Germany; Jorge.Hurtado.Oliveros@gmail.com (J.H.-O.); Jonathan.Wickel@med.uni-jena.de (J.W.); 2 Hans-Berger Department of Neurology, Jena University Hospital, 07747 Jena, Germany; 3 Department of Anesthesiology and Intensive Care, Jena University Hospital, 07747 Jena, Germany; cj.witt@gmx.de; 4 Department of Anesthesiology and Intensive Care Medicine, Center for Sepsis Control and Care (CSCC), and the Center for Molecular Biomedicine (CMB), Jena University Hospital, 07745 Jena, Germany; Markus.Graeler@med.uni-jena.de (M.H.G.); Ralf.Claus@med.uni-jena.de (R.A.C.); 5 Institute of Pharmacology and Toxicology, Jena University Hospital, 07747 Jena, Germany; Amelie.Lupp@med.uni-jena.de

**Keywords:** acid sphingomyelinase, ceramide, monooxygenase, functional inhibitors of sphingomyelin phosphodiesterase 1 (FIASMA), cytochrome P450, liver dysfunction, inflammation

## Abstract

Liver dysfunction during sepsis is an independent risk factor leading to increased mortality rates. Specifically, dysregulation of hepatic biotransformation capacity, especially of the cytochrome P450 (CYP) system, represents an important distress factor during host response. The activity of the conserved stress enzyme sphingomyelin phosphodiesterase 1 (SMPD1) has been shown to be elevated in sepsis patients, allowing for risk stratification. Therefore, the aim of the present study was to investigate whether SMPD1 activity has an impact on expression and activity of different hepatic CYP enzymes using an animal model of polymicrobial sepsis. Polymicrobial sepsis was induced in SMPD1 wild-type and heterozygous mice and hepatic ceramide content as well as CYP mRNA, protein expression and enzyme activities were assessed at two different time points, at 24 h, representing the acute phase, and at 28 days, representing the post-acute phase of host response. In the acute phase of sepsis, SMPD1^+/+^ mice showed an increased hepatic C16- as well as C18-ceramide content. In addition, a downregulation of CYP expression and activities was detected. In SMPD1^+/−^ mice, however, no noticeable changes of ceramide content and CYP expression and activities during sepsis could be observed. After 28 days, CYP expression and activities were normalized again in all study groups, whereas mRNA expression remained downregulated in SMPD^+/+^ animals. In conclusion, partial genetic inhibition of SMPD1 stabilizes hepatic ceramide content and improves hepatic monooxygenase function in the acute phase of polymicrobial sepsis. Since we were also able to show that the functional inhibitor of SMPD1, desipramine, ameliorates downregulation of CYP mRNA expression and activities in the acute phase of sepsis in wild-type mice, SMPD1 might be an interesting pharmacological target, which should be further investigated.

## 1. Introduction

Sepsis is characterized by an increasing incidence over the past 40 years currently ranging between 0.4/1000 and 1/1000 of the population. Additionally, it shows an unacceptably high mortality rate of almost 35% in intensive care units worldwide if not recognized early and treated promptly [1,2]. The liver is a central metabolic and immunologic organ, the function of which is impaired due to yet unclear molecular mechanisms during the course of the disease. It is composed of 60% parenchymal (hepatocytes) and 40% non-parenchymal cells (endothelial cells, hepatic stellate cells, Kupffer-cells), and these cells underlie complex interactions and adaptive mechanisms influencing metabolic and immunological capacity during host response [3].

Cytochrome P450 (CYP) enzymes are a family of heme proteins which are responsible for the (stepwise) metabolism of a plethora of exogenous (e.g., drugs) as well as endogenous low molecular weight compounds (e.g., steroids, bile acids) [4,5]. Seventeen gene families of mammalian CYP have been identified so far encoding for approximately 60 distinct CYP protein isoforms which are grouped according to their sequence similarity in eighteen groups and forty-four subgroups [6]. Of these, three enzyme groups (CYP families 1, 2 and 3) are estimated to be responsible for metabolizing >75% of drugs which are in clinical use at present. Considering the multitude of drugs simultaneously used in septic patients, detrimental effects on patients’ health due to alterations in CYP enzyme activity might go unnoticed [4]. Although many of the CYP enzymes are also present in extrahepatic organs, the highest concentrations are found in the liver, where it is bound to the endoplasmic reticulum [7]. It is well known that constituents of microorganisms such as endotoxin as well as pro-inflammatory cytokines released during host response can lead to a downregulation of CYP enzymes [8,9]. It could also be demonstrated that the induction of a systemic inflammatory condition in vivo in mice by peritoneal contamination and infection results in a reduction of hepatic CYP enzymes expression [10,11,12]. Likewise, in severely injured patients, decreased CYP activities were observed [13]. In addition, administration of endotoxin to healthy individuals resulted in an inhibition of CYP related drug metabolism [14], and biotransformation of atorvastatin was shown to be impaired in critically ill patients resulting in supra-therapeutic plasma concentrations [15].

In the last decades, acid sphingomyelinase has been repeatedly reported to play a major role in inflammatory host response and also in many liver diseases [16,17,18]. This enzyme is known to be an important regulator of many signal transduction pathways mediating processes such as cell differentiation, apoptosis or cell growth [19,20] and, therefore, might be an interesting target for therapies to overcome sepsis sequelae. Its hydrolysing activity results in the breakdown of inert, plasma membrane-embedded sphingomyelin to the highly active lipid mediator ceramide in the outer layer of cellular membranes. This process further leads to local ceramide accumulation in so called lipid rafts which is mediated by its biophysical properties [21]. The plasma activity of acid sphingomyelinase correlates with the severity of the disease and allows for the discrimination of patient outcomes [16,22,23]. In the course of endotoxemia and *Staphylococcus aureus* induced sepsis [24], functional inhibition of SMPD1 exerted protective effects and improved survival rates [16,25,26].

In the present study, we intended to investigate the impact of the stress responsive enzyme acid sphingomyelinase (SMPD1) on regulation of hepatic CYP expression and activities during sepsis using SMPD1 heterozygous animals relative to wild-type littermates.

## 2. Results

### 2.1. Parameters of Systemic Inflammation

**Figure 1:**
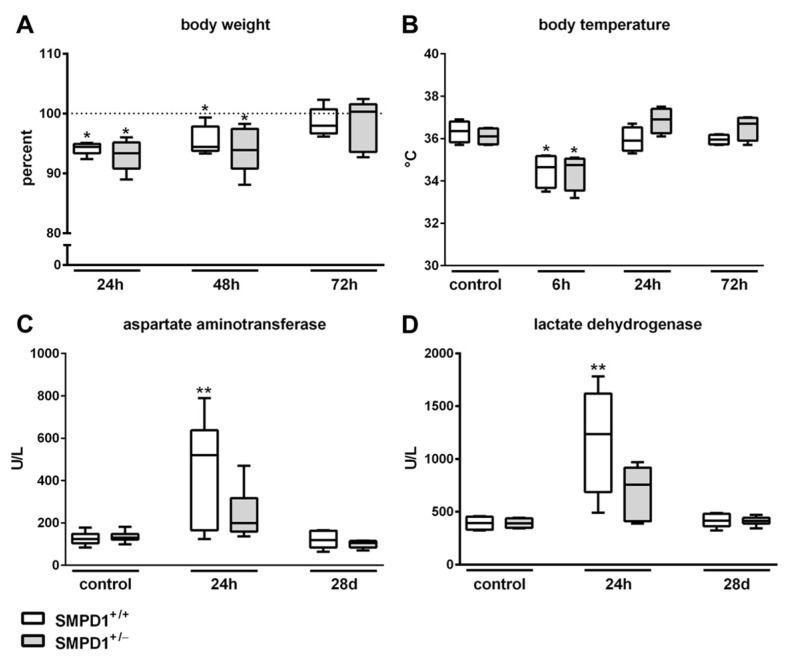
Measurement of markers for systemic inflammation. Body weight (**A**) and body temperature (**B**) were measured as non-invasive parameters of systemic inflammation at 24, 48 and 72 h following sepsis induction. (**C**,**D**) clinical markers for cell damage (aspartate aminotransferase, lactate dehydrogenase) were assessed in serum at baseline (control), 24 h as well as 28 days following polymicrobial sepsis induction. Data for all experiments were obtained from at least *n* ≥ 4 wild-type (SMPD1^+/+^) and heterozygous (SMPD1^+/−^) littermates. The dotted line (**A**) represents initial weight of animals prior to sepsis induction. * *p* ≤ 0.05 versus corresponding control; ** *p* ≤ 0.01 versus corresponding control (Mann-Whitney U test (MWU-test)).

To control the success of polymicrobial sepsis induction, non-invasive parameters were measured during the course of the systemic inflammation. Body weight dropped in wild-type animals to a median value of 94.44% (interquartile range (IQR) 25%: 93.38 and IQR 75%: 94.92%) of control values (100%) and in SMPD1 heterozygous littermates to a median value of 93.37% (IQR 25%: 90.81 and IQR 75%: 95.16%) 24 h following sepsis induction and was still significantly reduced at 48 h. At 72 h following sepsis induction, body weights had returned to normal values again (Figure 1A). Both animal groups showed a significant reduction of body temperature from a baseline median of 36.4 °C (SMPD1^+/+^) or 36.1 °C (SMPD1^+/−^), respectively, to a median of 34.7 °C (IQR 25%: 33.7 °C and IQR 75%: 35.2 °C) in wild-type or of 34.8 °C (IQR 25%: 33.6 °C and IQR 75%: 35.1 °C) in SMPD1^+/−^ animals, respectively, at 6 h following sepsis induction. At 24 h, values were normalized again (Figure 1B).

Regarding markers of cell damage in serum, aspartate aminotransferase (AST) showed an increase from 124.0 U/L (IQR 25%: 104.3 and IQR 75%: 148.3 U/L) to 519.5 U/L (IQR 25%: 165.0 and IQR 75%: 637.3 U/L) in wild-type animals 24 h following sepsis induction, whereas no significant effect could be detected in the respective SMPD^+/−^ littermates (control: 130.0 U/L (IQR 25%: 121.5 and IQR 75%: 148.0 U/L) vs. 24 h: 199.0 U/L (IQR 25%: 159.5 and IQR 75%: 316.5 U/L)) (Figure 1C). Similar results were observed when measuring the global cell injury marker lactate dehydrogenase (LDH). As can be seen from Figure 1D, significantly increased LDH levels were measured in the serum of wild-type animals (control: 392.5 U/L (IQR 25%: 330.3 and IQR 75%: 452.5 U/L) vs. 24 h: 1235.0 U/L (IQR 25%: 686.0 and IQR 75%: 1620.0 U/L)) 24 h following sepsis induction, whereas no respective elevation could be observed in the corresponding SMPD1^+/−^ littermates (control: 391.5 U/L (IQR 25%: 347.8 and IQR 75%: 438.3 U/L) vs. 24 h: 757.0 U/L (IQR 25%: 410.0 and IQR 75%: 918.0 U/L)).

### 2.2. Sphingomyelin Phosphodiesterase 1 (SMPD1) Triggered Changes in the Composition of Hepatic Ceramides Following Polymicrobial Sepsis Induction

**Figure 2:**
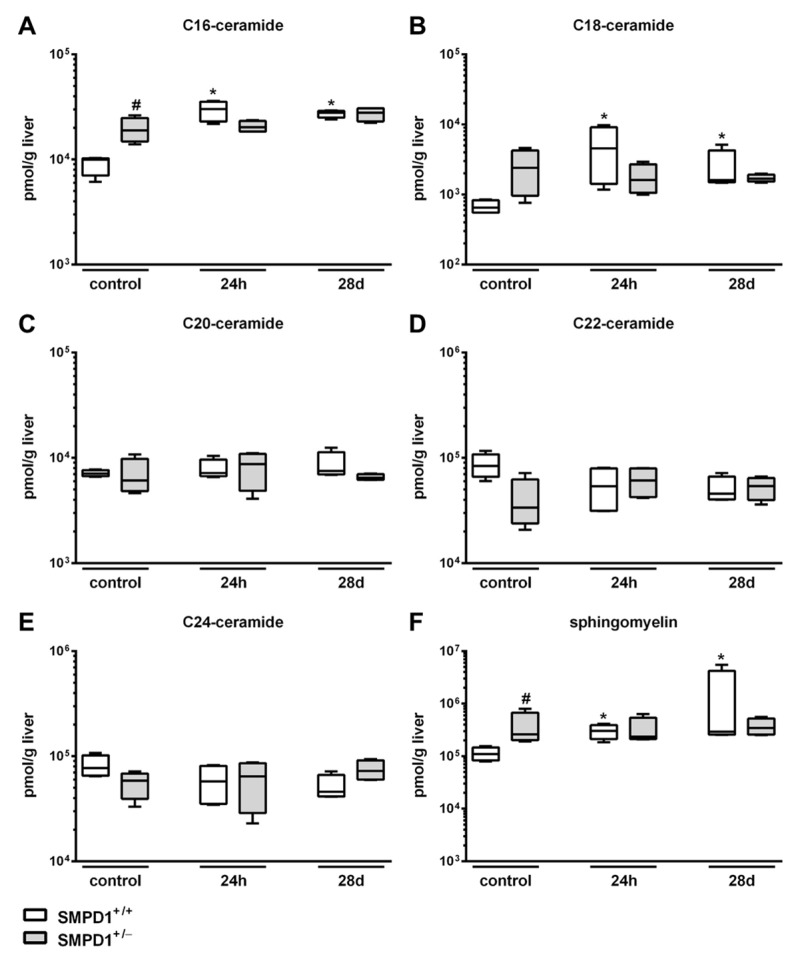
Increased hepatic ceramide levels in SMPD1^+/+^ mice following polymicrobial sepsis induction. C16-ceramide (**A**), C18-ceramide (**B**), C20-ceramide (**C**), C22-ceramide (**D**), C24-ceramide (**E**) and sphingomyelin (**F**) content was measured in liver tissue homogenates by mass spectrometry at baseline (control; *n* = 4), 24 h and 28 days following polymicrobial sepsis induction (SMPD1^+/+^, SMPD1^+/−^; *n* = 4 each). * *p* ≤ 0.05 versus corresponding control; # *p* ≤ 0.05 versus corresponding SMPD1^+/+^ animals (MWU-test).

Ceramide is known to be generated through different pathways; however, SMPD1 seems to be the key enzyme in the rapid and transient generation of this mediator [27,28]. In SMPD1^+/+^ animals, an enhancement of C16-ceramide generation (Figure 2A) could be observed from a median control level of 9944 (IQR 25%: 7026 and IQR 75%: 10,240) pmol/g liver to 30,165 (IQR 25%: 22,916 and IQR 75%: 35,492) pmol/g liver 24 h following septic insult. However, the increase of C16-ceramide content (27,780 (IQR 25%: 24,999 and IQR 75%: 28,910) pmol/g liver) remained significantly elevated at 28 days following sepsis induction as compared to corresponding baseline (control) animals. In comparison to wild-type mice, SMPD1^+/−^ animals showed significantly higher baseline values of C16-ceramide (18,891 (IQR 25%: 14,829 and IQR 75%: 24,764) pmol/g liver). These levels remained stable over time following polymicrobial sepsis. Similarly, in SMPD1^+/+^ animals, C18-ceramide levels (Figure 2B) increased significantly from a median value of 651 (IQR 25%: 550 and IQR 75%: 825) pmol/g liver to 4544 (IQR 25%: 1419 and IQR 75%: 9053) pmol/g liver at 24 h and to 1590 (IQR 25%: 1495 and IQR 75%: 4253) pmol/g liver at day 28 following septic insult. Levels in SMPD1^+/−^ animals remained stable over time with higher levels at baseline as compared to the respective SMPD1^+/+^ littermates. In contrast, C20-, C22- as well as C24-ceramide levels showed no changes during sepsis in both groups of animals (Figure 2C–E). In SMPD1^+/−^ mice, the high levels of C16- and C18-ceramide at baseline were mirrored by significantly higher values of sphingomyelin at baseline (control: 261,616 (IQR 25%: 201,339 and IQR 75%: 674,209) pmol/g liver) as compared to corresponding SMPD1^+/+^ animals (control: 109,074 (IQR 25%: 82,887 and IQR 75%: 146,994) pmol/g liver; Figure 2F). Interestingly, the amount of sphingomyelin in liver tissue increased in SMPD1^+/+^ animals from a median value of 109,074 (IQR 25%: 82,887 and IQR 75%: 146,994) pmol/g liver to a median level of 302,121 (IQR 25%: 211,177 and IQR 75%: 390,042) pmol/g liver at 24 h and remained high at day 28 following septic insult.

### 2.3. Hepatic Cytochrome P450 mRNA Expression Following Polymicrobial Sepsis Induction

**Figure 3:**
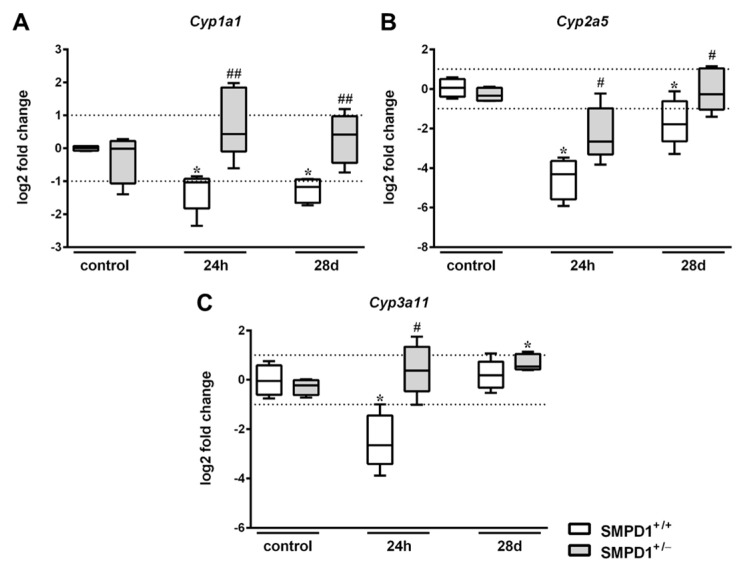
Hepatic cytochrome P450 mRNA expression following polymicrobial sepsis induction. Quantitative real-time PCR of (**A**) *Cyp1a1*, (**B**) *Cyp2a5*, (**C**) *Cyp3a11* was performed in liver tissue of wild-type (SMPD1^+/+^) as well as of heterozygous (SMPD1^+/−^) littermates at baseline (control), at 24 h and at 28 days following polymicrobial sepsis induction. The method of Pfaffl [29] was used to normalize (unvaried reference transcript: *Actb*) and to calculate the expression levels of the transcripts. Box plots are presented in log2 fold changes. Cut-off values were set at ±1, representing a variation of biological significance (dotted line). Control: *n* = 4, 24 h: *n* = 5, 28 days: *n* = 5. Levels of significance between control and septic groups are indicated by asterisks (* *p* ≤ 0.05; MWU-test) and between SMPD1^+/−^ and SMPD1^+/+^ littermates at the same time point by a rhombus (# *p* ≤ 0.05; ## *p* ≤ 0.01; MWU-test).

The expression rate of *Cyp1a1* in SMPD1^+/+^ animals significantly decreased from a median control level set to 0.0 (IQR 25%: −0.08 and IQR 75%: 0.07) log2 fold change to −1.04 (IQR 25%: −1.83 and IQR 75%: −0.93) log2 fold change at 24 h following polymicrobial sepsis induction (Figure 3A). In contrast, partial SMPD1 deficiency in heterozygous littermates (SMPD1^+/−^) revealed a median value of 0.43 log2 fold change after sepsis induction (IQR 25%: −0.10 and IQR 75%: 1.85 log2 fold change), which was significantly increased in comparison to the control situation, but also significantly higher as compared to SMPD1^+/+^ mice following sepsis induction. As displayed in Figure 3B, induction of peritoneal sepsis resulted in the reduction of *Cyp2a5* expression in SMPD1^+/+^ mice (control: 0.06 (IQR 25%: −0.4 and IQR 75%: 0.49) log2 fold change vs. 24 h: −4.32 (IQR 25%: −1.83 and IQR 75%: −0.93) log2 fold change), whereas SMPD1^+/−^ animals (−2.66 (IQR 25%: −3.31 and IQR 75%: −0.98) log2 fold change; *p* ≤ 0.05) demonstrated a significantly less pronounced reduction of the values following sepsis induction as compared to SMPD1^+/+^ littermates. In addition, *Cyp3a11* expression (Figure 3C) showed a significant decrease of the median value in SMPD1^+/+^ animals from −0.05 (IQR 25%: −0.61 and IQR 75%: 0.59) log2 fold change to −2.65 (IQR 25%: −3.41 and IQR 75%: −1.45) log2 fold change following sepsis induction. Induction of polymicrobial sepsis in SMPD1^+/−^ littermates, in contrast, resulted in a diminished reduction of expression levels as compared to SMPD1^+/+^ mice (0.38 (IQR 25%: −0.47 and IQR 75%: 1.34) log2 fold change; *p* ≤ 0.05).

After 28 days, hepatic *Cyp1a1* expression rate in SMPD1^+/+^ mice still remained downregulated (−1.17 (IQR 25%: −1.66 and IQR 75%: −0.95) log2 fold change), whereas the expression rate in SMPD1^+/−^ littermates was found to be significantly higher as compared to the corresponding septic wild-type animals (0.41 (IQR 25%: −0.45 and IQR 75%: 0.97) log2 fold change) (Figure 3A). In addition, hepatic *Cyp2a5* expression was still found to be downregulated in SMPD1^+/+^ animals (−1.79 (IQR 25%: −2.65 and IQR 75%: −0.62) log2 fold change) at 28 days following polymicrobial sepsis, whereas heterozygous animals, again, demonstrated significantly improved values in comparison to septic wild-type mice (−0.27 (IQR 25%: −1.04 and IQR 75%: 1.04) log2 fold change) (Figure 3B). *Cyp3a11* expression, in contrast, was not altered both in wild-type as well as in heterozygous survivors of polymicrobial sepsis at day 28 (Figure 3C).

### 2.4. Hepatic Cytochrome P450 Activity Following Polymicrobial Sepsis Induction

**Figure 4:**
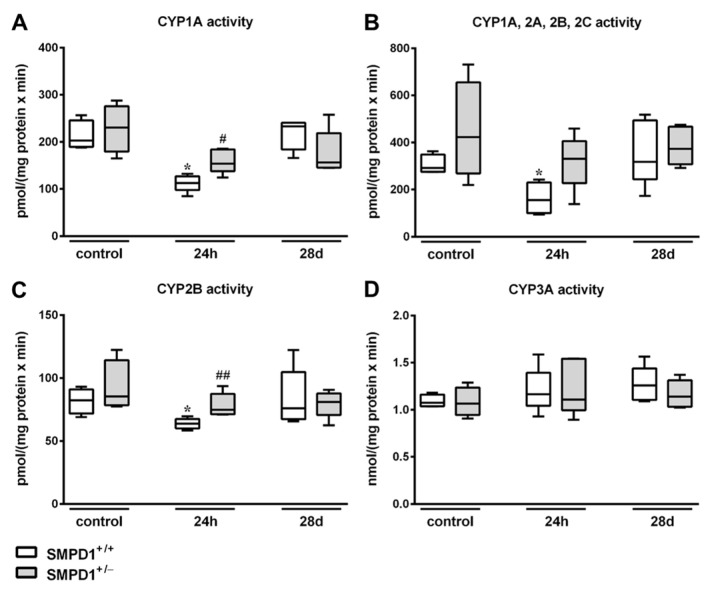
Hepatic monooxygenase activities following polymicrobial sepsis induction. Activity of (**A**) CYP1A (ethoxyresorufin-*O*-deethylation; EROD), (**B**) CYP1A, 2A, 2B, 2C (ethoxycoumarin-*O*-deethylation; ECOD), (**C**) CYP2B (pentoxyresorufin-*O*-depentylation; PROD), (**D**) CYP3A (ethylmorphine-*N*-demethylation; EMND) was measured in liver tissue 9000× *g* supernatants from SMPD1^+/+^ as well as from SMPD1^+/−^ mice at baseline (control), at 24 h and at 28 days following sepsis induction. Data were obtained from *n* = 4 wild-type (SMPD1^+/+^) and heterozygous (SMPD1^+/−^) littermates at each time point. Levels of significance between control and septic groups are indicated by asterisks (* *p* ≤ 0.05; MWU-test) and between SMPD1^+/−^ and SMPD1^+/+^ littermates at the same time point by a rhombus (# *p* ≤ 0.05; ## *p* ≤ 0.01; MWU-test).

Hepatic CYP1A activities, as assessed by EROD (Figure 4A), showed a significant reduction of the values in SMPD1^+/+^ animals 24 h after sepsis induction from a median value of 203.3 (IQR 25%: 189.5 and IQR 75%: 245.6) pmol/(mg protein × min) to 112.5 (IQR 25%: 97.8 and IQR 75%: 126.9) pmol/(mg protein × min). In contrast, SMPD1^+/−^ mice (control: 230.7 (IQR 25%: 179.3 and IQR 75%: 275.7) pmol/(mg protein × min) vs. 24 h: 153.9 (IQR 25%: 138.1 and IQR 75%: 184.0) pmol/(mg protein × min)) demonstrated a significantly smaller decline in CYP1A activity 24 h following sepsis induction. Similar results were detected using ECOD, measuring CYP1A, 2A, 2B, 2C activities (Figure 4B). At 24 h after sepsis induction, SMPD1^+/+^ animals displayed a significant decrease in enzyme activities (control: 291.9 (IQR 25%: 275.5 and IQR 75%: 348.8) pmol/(mg protein × min) vs. 24 h: 155.5 (IQR 25%: 99.2 and IQR 75%: 230.1) pmol/(mg protein × min); *p* ≤ 0.05). In SMPD1^+/−^ littermates, in contrast, no significant influence of sepsis induction on enzyme activities could be observed (331.3 (IQR 25%: 227.5 and IQR 75%: 405.8) pmol/(mg protein × min)) as compared to the corresponding control group (422.6 (IQR 25%: 268.9 and IQR 75%: 655.5) pmol/(mg protein × min)). Likewise, CYP2B activity as assessed by PROD (Figure 4C) was reduced from 82.3 (IQR 25%: 71.8 and IQR 75%: 90.9) pmol/(mg protein × min) to 63.7 (IQR 25%: 60.0 and IQR 75%: 67.5) pmol/(mg protein × min) (*p* ≤ 0.05) in SMPD1^+/+^ animals, whereas SMPD1^+/−^ littermates (control: 85.3 (IQR 25%: 78.4 and IQR 75%: 114.2) pmol/(mg protein × min) vs. 24 h: 74.9 (IQR 25%: 71.2 and IQR 75%: 87.4) pmol/(mg protein × min)) showed no reduction of activities after sepsis induction and, consequently, significantly higher levels as compared to their respective septic wild-type mice (*p* ≤ 0.05). Interestingly, the activity of CYP3A, as measured by EMND (Figure 4D), was not affected during host response in both SMPD1^+/+^ as well as SMPD1^+/−^ animals.

All CYP enzymes tested (Figure 4A–D) showed normalized activity values in the long-term following polymicrobial sepsis induction as compared to the control situation and there were no differences between the values of the wild-type and the heterozygous animals.

### 2.5. Hepatic Cytochrome P450 Isoforms Expression after Polymicrobial Sepsis Induction

**Figure 5:**
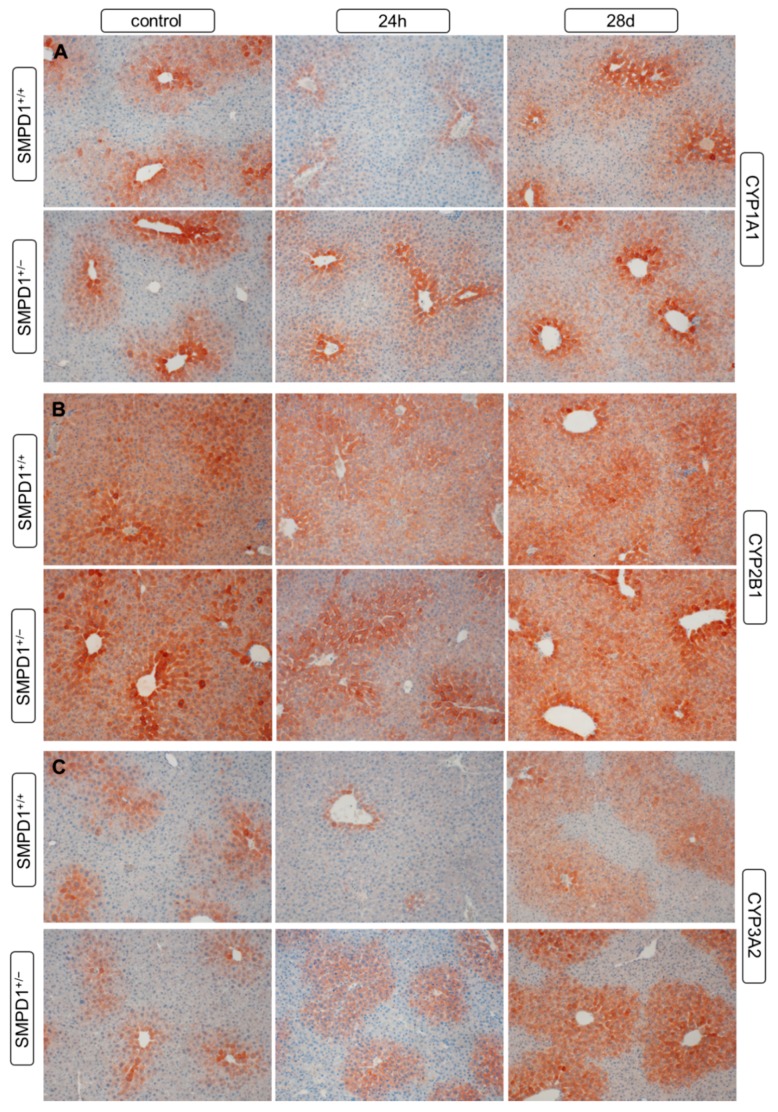
Hepatic cytochrome P450 isoforms expression following polymicrobial sepsis induction. Expression of hepatic monooxygenases ((**A**) CYP1A1, (**B**) CYP2B1, (**C**) CYP3A2) was determined using immunohistochemistry (red-brown color, counterstaining with hematoxylin) in SMPD1^+/+^ as well as in SMPD1^+/−^ mice at baseline (control), at 24 h and at 28 days following sepsis induction. Representative photomicrographs are shown from *n* = 4 different liver tissue samples (original magnification: 200×).

All three CYP isoforms, CYP1A (Figure 5A), CYP2B (Figure 5B) as well as CYP3A (Figure 5C), assessed showed a predominant expression around the central veins and in the intermediate regions of the liver lobules, with no major differences in the expression patterns between SMPD^+/+^ and SMPD^+/−^ animals. In comparison to the control situation, 24 h after sepsis induction in SMPD^+/+^ animals CYP expression was distinctly reduced. In SMPD^+/−^ mice, in contrast, no noticeable change in the expression pattern was seen.

At 28 days after sepsis induction, in both groups of mice, no differences were detectable in the hepatic CYP expression patterns between sepsis survivors and respective control animals. There were also no major differences between SMPD1^+/+^ and SMPD1^+/−^ mice.

### 2.6. Functional Inhibition of SMPD1 with Desipramine Improves Transcriptional Expression of Hepatic Monooxygenases during Host Response

**Figure 6:**
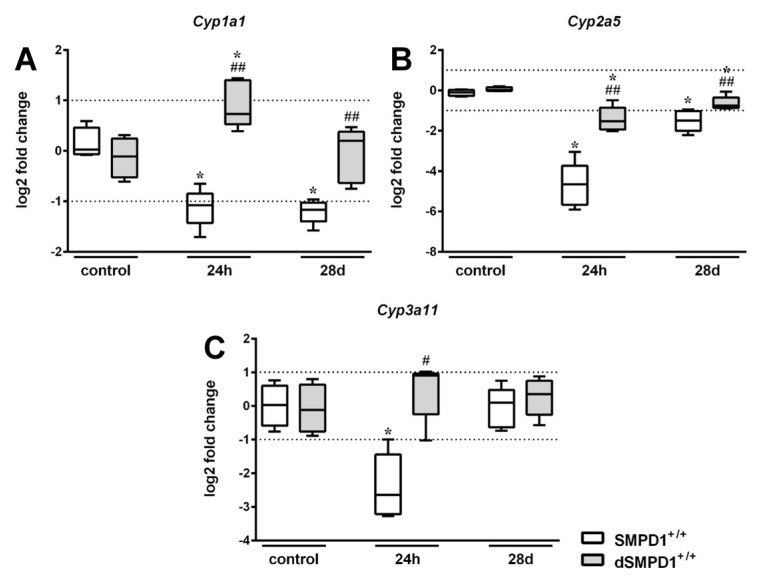
Influence of the functional SMPD1 inhibitor desipramine on hepatic CYP mRNA expression following polymcirobial sepsis induction. Quantitative real-time PCR of (**A**) *Cyp1a1*, (**B**) *Cyp2a5*, (**C**) *Cyp3a11* was performed in liver tissue of wild-type (SMPD1^+/+^) as well of desipramine pretreated wild-type littermates (dSMPD1^+/+^) at baseline (control *n* = 4), at 24 h and at 28 days following polymicrobial sepsis induction (*n* = 5). The method of Pfaffl [29] was used to normalize (unvaried reference transcript: *Actb*) and to calculate the expression levels of the transcripts. Box plots are presented in log2 fold changes. Cut-off values were set at ±1, representing a variation of biological significance (dotted line). Levels of significance between control and septic groups are indicated by asterisks (* *p* ≤ 0.05; MWU-test) and between dSMPD1^+/+^ and SMPD1^+/+^ littermates at the same time point by a rhombus (# *p* ≤ 0.05; ## *p* ≤ 0.01; MWU-test).

The transcriptional expression of (A) *Cyp1a1*, (B) *Cyp2a5*, (C) *Cyp3a11* was significantly downregulated in SMPD1^+/+^ mice at 24 h following sepsis induction (Figure 6A–C). In desipramine pretreated littermates (dSMPD1^+/+^), however, this decrease in CYP mRNA expression was distinctly less pronounced. *Cyp1a1* expression in dSMPD1^+/+^ mice amounted to a median value of 0.73 (IQR 25%: 0.53 and IQR 75%: 1.40) log2 fold change, which was significantly higher as compared to the respective control animals (−0.11 (IQR 25%: −0.53 and IQR 75%: 0.25)) and to the SMPD1^+/+^ littermates at 24 h following sepsis induction (−1.08 (IQR 25%: −1.43 and IQR 75%: −0.85); Figure 6A). Similarly, the transcriptional expression of *Cyp2a5* (−1.53 (IQR 25%: −1.94 and IQR 75%: −0.86) log2 fold change) as well as *Cyp3a11* (0.90 (IQR 25%: −0.25 and IQR 75%: 0.96) log2 fold change) showed higher median values in dSMPD1^+/+^ mice as compared to the SMPD1^+/+^ littermates at 24 h following sepsis induction (*Cyp2a5*: −4.65 (IQR 25%: −5.67 and IQR 75%: −3.74); *Cyp3a11* (−2.65 (IQR 25%: −3.22 and IQR 75%: −1.45)). Indeed, in dSMPD1^+/+^ mice *Cyp2a5* values were only slightly reduced and *Cyp3a11* values did even not significantly differ from those of the respective desipramine treated control animals at baseline (*Cyp2a5*: 0.25 (IQR 25%: −0.03 and IQR 75%: 0.15); *Cyp3a11*: −0.12 (IQR 25%: −0.76 and IQR 75%: 0.64); Figure 6B,C).

As described above (Figure 3A–C), transcriptional expression of *Cyp1a1* and *Cyp2a5* was still significantly downregulated in SMPD1^+/+^ animals at 28 days following sepsis induction, whereas treating SMPD1^+/+^ littermates with desipramine resulted in improved *Cyp1a1* (0.20 (IQR 25%: −0.64 and IQR 75%: 0.38) log2 fold change) and *Cyp2a5* (−0.76 (IQR 25%: −0.88 and IQR 75%: −0.36) log2 fold change) expression rates (Figure 6A,B).

### 2.7. Functional Inhibition of SMPD1 with Desipramine Improves Hepatic Monooxygenase Activities during Host Response

**Figure 7:**
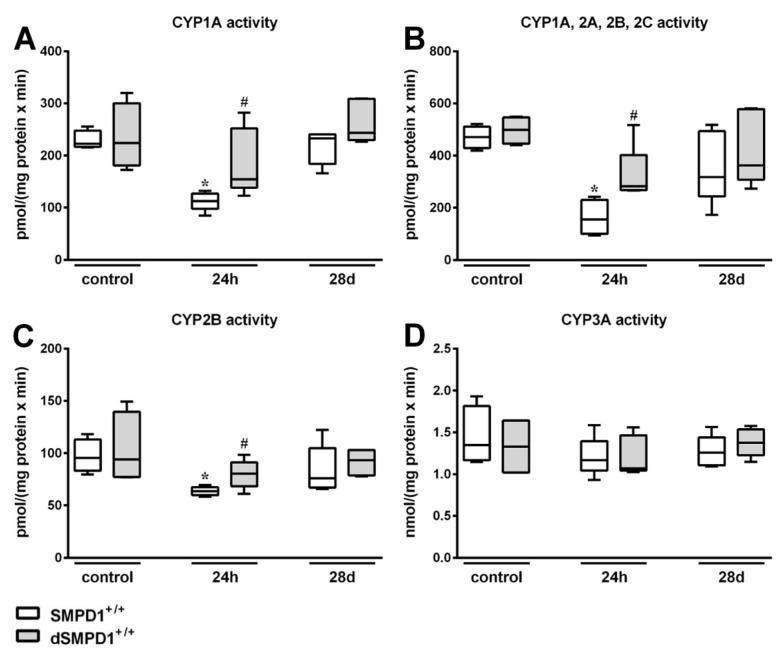
Influence of the functional SMPD1 inhibitor desipramine on hepatic monooxygenase activities following polymicrobial sepsis induction. Activity of (**A**) CYP1A (ethoxyresorufin-*O*-deethylation; EROD), (**B**) CYP1A, 2A, 2B, 2C (ethoxycoumarin-*O*-deethylation; ECOD), (**C**) CYP2B (pentoxyresorufin-*O*-depentylation; PROD) and (**D**) CYP3A (ethylmorphine-*N*-demethylation; EMND) was measured in liver tissue 9000× *g* supernatants of wild type (SMPD1^+/+^) as well as of desipramine pretreated wild-type littermates (dSMPD1^+/+^) at baseline (control; *n* = 4), at 24 h and at 28 days following sepsis induction (*n* = 5). Levels of significance between control and septic groups are indicated by asterisks (* *p* ≤ 0.05; MWU-test) and between dSMPD1^+/+^ and SMPD1^+/+^ littermates at the same time point by a rhombus (# *p* ≤ 0.05; MWU-test).

In the livers of the vehicle-only treated mice, with the only exception of EMND, all CYP model reactions tested showed a distinct reduction in the activities at 24 h after polymicrobial sepsis induction. This reduction in CYP activities, in contrast, was significantly less pronounced in the desipramine pretreated animals and the values did even not significantly differ from those of the respective control mice. At 24 h following sepsis induction in desipramine pretreated SMPD1^+/+^ animals median CYP1A activities (EROD; Figure 7A) amounted to 154.4 (IQR 25%: 138.6 and IQR 75%: 252.1) pmol/(mg protein × min), median CYP1A, 2A, 2B and 2C activities (ECOD; Figure 7B) to 282.4 (IQR 25%: 268.4 and IQR 75%: 402.4) pmol/(mg protein × min) and median CYP2B activities (PROD; Figure 7C) to 80.4 (IQR 25%: 68.4 and IQR 75%: 91.2) pmol/(mg protein × min). The median values of the respective controls at baseline were 224.3 (IQR 25%: 180.9 and IQR 75%: 300.5) pmol/(mg protein × min) for CYP1A activities, 499.2 (IQR 25%: 446.0 and IQR 75%: 545.9) pmol/(mg protein × min) for CYP1A, 2A, 2B and 2C activities, and 93.9 (IQR 25%: 77.2 and IQR 75%: 139.5) pmol/(mg protein × min) for CYP2B activities (Figure 7A–D).

All CYP enzymes tested (Figure 7A–D) showed normalized activity values at 28 days following sepsis induction with no differences between vehicle treated SMPD1^+/+^ and desipramine pretreated SMPD1^+/+^ animals.

## 3. Discussion

Sepsis is still a major health problem in intensive care worldwide. At present, there is no specific treatment available which is based on the underlying molecular mechanisms. Therefore, therapy options are thus far limited to early and appropriate antibiotic treatment, volume resuscitation and vasopressor administration [30]. Clinical studies with promising drug candidates such as antithrombin III [31] or human recombinant activated protein C have failed so far [30,32]. In particular, hepatic dysfunction is an independent predictor of patient mortality during severe systemic inflammation [33]. During septic shock, impairment of hepatic blood flow results in hypoperfusion of liver tissue causing hypoxic hepatitis. Furthermore, alterations in the bile transporter systems of the hepatocytes result in excretory dysfunction, hyperbilirubinemia and cholestasis [34]. Polymedication of patients during their stay in the intensive care units further increases the risk of liver injury [35]. In sepsis, hepatic activity of numerous CYPs is distinctly reduced and this effect might contribute to multi-organ failure and ultimately death [7,12,13]. It is well known that pro-inflammatory cytokines, such as TNF-α, IL-1β, IFN-γ or IL-6, are capable of downregulating the expression of different hepatic CYP isoforms [7,9,11]. Their essential role in sepsis is also demonstrated by an exacerbated pro-inflammatory response and an increased mortality rate following pharmacological inhibition of monooxygenases in an animal model of systemic inflammation using zymosan injection [36]. In human volunteers, injection of endotoxin resulted in a significant suppression of CYP activity, which additionally correlated to the degree of circulating IL-6 in plasma in these individuals [14]. In a previous study, we were already able to demonstrate that partial genetic inihibition of the conserved stress responsive enzyme acid sphingomyelinase results in an improved hepatobiliary function, diminished hepatic pro-inflammatory response and reduced hepatic stellate cell activation during the course of systemic inflammation [25]. In the present study, as an immediate surrogate for increased SMPD1 activity, liver tissue levels of C16- and C18-ceramide were increased in wild-type animals after sepsis induction, reflecting cellular stress response, whereas levels of C20-, C22- and C24-ceramide remained stable. It is well known that the distribution of sterols within sphingomyelin-containing membranes and the addition/generation of ceramides affect sterol partitioning in a chain length dependent manner and that ceramides with intermediate chain lengths (i.e., C16-, C18-ceramides) are the most effective in reducing sterol partitioning into the membrane influencing its lateral organization and forming separate ceramide-enriched domains [37,38]. Isolated cells lacking SMPD1 have been shown to be protected against Fas-ligand and TNF-α mediated apoptosis, whereas treating these cells with C16-ceramide restored the capability of generating ceramide-enriched rafts resulting in cell death [39,40]. These results suggest a direct correlation between SMPD1 activity and C16-ceramide generation. Less is known in the case of SMPD1 and C18-ceramide. Furthermore, a distinct accumulation of sphingomyelin within the cell membrane has been already described for SMPD1 knock-out animals [41]. Regarding our findings of increased baseline levels of C16- and C18-ceramide in heterozygous littermates, we suggest a compensating accumulation on the basis of elevated baseline levels of sphinogmyelin and decreased levels of C22- and C24-ceramide. However, ceramide content did not further increase following septic insult. This stabilization of hepatic ceramide content results from a lack of increase in SMPD1 activity during host response, as we have demonstrated recently [25]. From these data, we conclude that modifications in lipid composition of the cell membrane, such as an increase of ceramide content, and changes in ceramide/sphingomyelin ratio following a biological stimulus are decisive rather than the baseline content of ceramide.

Regarding liver biotransformation capacity, our present study revealed that the activity as well as the mRNA and protein expression of selected and important CYP enzymes were less impaired in SMPD1^+/−^ mice at 24 h after induction of polymicrobial infection as compared to wild-type animals. The involvement of SMPD1 in CYP regulation is confirmed by other groups showing that stimulation with C2-ceramide or exogenous acid sphingomyelinase in rat hepatocytes results in a downregulation of CYP2A11 mRNA and protein expression [42]. Furthermore, sepsis-associated pro-inflammatory cytokines, such as TNF-α, induce activation of SMPD1 and, subsequently, the formation of ceramide in primary isolated hepatocytes [43]. Fittingly, we were already able to demonstrate that SMPD1 heterozygous animals show less pronounced hepatic cytokine expression, which in turn might result in stabilization of SMPD1 activity as well as hepatic ceramide content and, therefore, inhibition of monooxygenase downregulation during sepsis [25]. To the best of our knowledge, the present study is the first to describe a direct association between acid sphingomyelinase activities, hepatic ceramide levels and alterations in the transcriptional and protein expression levels as well as enzyme activities of different CYPs in the acute phase of sepsis. Both, a better understanding of underlying mechanisms regulating pharmacokinetic behaviour of drugs [10,34,44] as well as strategies for individualisation and optimization of dosage, are urgently needed [45,46] in order to face pharmacokinetic variability in sepsis patients due to complex overlap of multiple pathophysiological and clinical factors and to prevent unpredictable exposure from standard dosage regimens influencing patient outcome. The present data are of major clinical interest since numerous drugs used in daily clinical practice exert a partial inhibitory effect on SMPD1 activity. These so called functional inhibitors of SMPD1 (FIASMA) comprise a broad range of substances, which are frequently prescribed for psychiatric disease entities: desipramine, imipramine, fluphenazine, fluoxetine and amitriptyline, to mention only a few [47]. Their amphiphilic character leads to their diffusion into the cytoplasm of cells and their cationic nature to their accumulation in lysosomes, where they promote the proteolysis of SMPD1, thus decreasing its activity by almost 60–80%. These drugs have already been shown to reduce mortality of mice during endotoxemia and polymicrobial sepsis [16,25], an effect which might be attributed to their anti-inflammatory character by inhibiting immune cell activation [48,49].

Besides acute alterations, peritoneal sepsis has been demonstrated to initiate remodeling of hepatic parenchyma both in humans [50] as well as in a long-term murine model of polymicrobial sepsis [51]. In the present investigation, no alterations of CYP enzyme activity and protein expression could be detected in the post-acute phase of sepsis (28 days) in both animal groups. Transcriptional expression rate on the other hand was still downregulated in SMPD1^+/+^ mice, confirming a long-term impact of polymirobial sepsis on hepatic function in sepsis survivors. Again, this effect was less pronounced in SMPD1^+/−^ littermates.

Based on previous findings of our group showing a beneficial effect of the FIASMA desipramine on hepatic cytokine expression, oxidative stress, cholestasis and fibrotic changes in polymicrobial sepsis [25] and as a proof of concept for the present results, in the present study, we also tested the effect of this drug in SMPD1 wild-type mice on hepatic CYP mRNA expression and on monooxygenase activities following polymicrobial sepsis induction. As to be expected and confirming these previous findings, desipramine exerted a clear-cut protective effect also on this liver function parameter.

In summary, in the present study, we were able to show that the inhibition of the stress responsive enzyme acid sphingomyelinase stabilizes hepatic ceramide content and improves hepatic CYP activity during the acute phase of polymicrobial sepsis. Based on these results, in a translational approach, sepsis patients medicated with functional SMPD1 inhibitors due to other medical reasons could be identified and compared to those which are not treated with these drugs regarding parameters such as outcome, days of ventilation and organ dysfunction. Moreover, researchers investigating sepsis patients should take the effects of polymedication into account, including FIASMAs, which might lead to unexpected results because of their anti-inflammatory capacity and, therefore, may lead to a misinterpretation of the results.

## 4. Material and Methods

### 4.1. Animals and Sepsis Model

All experiments were performed in accordance with the German legislation on protection of animals and with the approval of the respective animal welfare committee (Thueringer Landesamt fuer Lebensmittelsicherheit und Verbraucherschutz, approval No.: 02-009/12). For each experiment, similar proportions of male and female mice from SMPD1^+/−^ as well as SMPD1^+/+^ littermates were randomly selected at the age of 8–12 weeks (mean body weight (B.W.): 21.7 g). Animals were housed under standardized conditions as described previously [25]. For induction of abdominal sepsis, a standardized peritoneal contamination and infection protocol was used, injecting human-derived fecal slurry (3 µL/g body weight) into the right lower quadrant of the abdomen with a 21-gauge cannula [52]. Following sepsis induction, a standard regime of antibiotics and volume resuscitation (20 mg/kg B.W. meropenem every 24 h subcutaneously (s.c.) and 25 µL/g B.W. physiological saline solution s.c. twice daily over 4 days, starting 6 h following sepsis induction) were administered and led to a survival rate of 60%. At defined time points, body temperature and body weight were measured. The Clinical Severity Score (CSS) was used to assess the condition of the mice every three hours following sepsis induction, as described previously [52]. Animals (*n* ≥ 4 per group) were sacrificed at baseline (control), at 24 h as well as at 28 days following sepsis induction. Livers were then excised and either fixed in 10% buffered formalin overnight and subsequently embedded in paraffin blocks or snap-frozen in liquid nitrogen and stored at −80 °C until biochemical analysis. It is known from the literature that, as soon as the animals have reached the age of two months, the CYP expression pattern and activities in livers of mice and rats do not change significantly up to senescence (18- to 24-month-old animals) [53,54,55,56,57]. Therefore, for ethical reasons, we have renounced a separate group of age-matched control animals at 28 days after sepsis induction.

For the investigation of the effects of the functional SMPD1 inhibitor desipramine on polymicrobial sepsis-induced systemic inflammation, randomly selected SMPD1^+/+^ animals were treated for seven days with desipramine hydrochloride (20 mg/kg body weight, dissolved in 0.9% NaCl) every 24 h subcutaneously prior to induction of polymicrobial infection, which was continued up to euthanasia. Respective non-desipramine-treated animals received the respective vehicle only (0.9% NaCl). Animals (*n* ≥ 4 per group) were sacrificed at baseline (control), at 24 h and at 28 days after polymicrobial sepsis induction.

### 4.2. Laboratory Markers

During the course of the systemic inflammation, clinically approved markers of cell damage—aspartate aminotransferase (AST) and lactate dehydrogenase (LDH)—were analysed in serum of wild-type (*n* = 8) as well as SMPD1 heterozygous littermates (*n* = 8) at baseline (control), 24 h and 28 days following sepsis induction using the clinical chemistry analyser Fuji Dri-Chem 3500i (Sysmex, Leipzig, Germany) according to manufacturer’s instructions.

### 4.3. Mass Spectrometry

Fresh frozen liver tissue from each stratum (SMPD1^+/+^ and SMPD1^+/−^; *n* = 4) at baseline (control), 24 h and 28 days following septic insult was analyzed with respect to C16-, C18-, C20-, C22-, C24-ceramide and sphingomyelin content. Measurements were performed according to an established protocol using liquid chromatography coupled to triple-quadrupole mass spectrometry [58]. For detection, the QTrap triple-quadrupole mass spectrometer (ABSciex, Darmstadt, Germany) interfaced with the Merck-Hitachi Elite LaChrom series 3.1.3 chromatograph (Düsseldorf, Germany) and autosampler (VWR) was used and analyzed using Analyst 1.4 (ABSciex).

### 4.4. Real-Time PCR

Liver tissue samples (15–20 mg) from control or animals following polymicrobial sepsis (*n* = 4), were homogenized in 600 µL lysis buffer (RLT lysis buffer, supplemented with 1% β-mercaptoethanol; Qiagen, Hilden, Germany). RNA was isolated using RNeasy Mini Kit according to manufacturer’s instructions (Qiagen, Hilden, Germany). RNA concentration and integrity were analyzed using a Nanodrop™ spectrophometer and QIAxcel microcapillar electrophoresis apparatus, which was followed by reverse transcription of 1.0 µg mRNA using standard conditions (Thermo Scientific, Dreieich, Germany) [59]. The mRNA expression profile of selected genes was measured using a Rotor-Gene Q 2plex system. The following primer sequences were used:
*Cyp1a1*forward:TGAGAAGGGCCACATCCGGreverse:CCAAAGAGGTCCAAAACAATCG*Cyp2a5*forward:ATCGGGTGATTGGCAGGAACreverse:TGGGGATCATGTCTGCAAATCT*Cyp3a11*forward:AGCAGGGATGGACCTGGreverse:CGGTAGAGGAGCACCAA*Actb*forward:GCTCTTTTCCAGCCTTCCTTreverse:CGGATGTCAACGTCACACTT

### 4.5. Immunohistochemistry

For immunohistochemical analysis of CYP isoforms expression, 3-μm sections were prepared from the paraffin blocks and floated onto positively charged slides. Immunostaining was performed by an indirect peroxidase-labeling method, as described previously [60]. Briefly, sections were de-waxed, microwaved in 10 mM citric acid (pH 6.0) for 16 min at 600 W, and incubated with the respective primary antibodies (goat anti-rat CYP1A1, CYP2B1/2 or CYP3A2, Daiichi Pure Chemicals, Tokyo, Japan; dilution: 1:5000) at 4 °C overnight. Detection of the primary antibody was performed using a biotinylated rabbit anti-goat IgG, followed by incubation with peroxidase-conjugated avidin (Vector ABC “Elite” kit, Vector, Burlingame, CA, USA). Binding of the primary antibody was visualized using 3-amino-9-ethylcarbazole (AEC) in acetate buffer (BioGenex, San Ramon, CA, USA). The sections were then rinsed, counterstained with Mayer’s hematoxylin (Sigma Aldrich, Steinheim, Germany), and mounted in Vectamount™ mounting medium (Vector Laboratories, Burlingame, CA, USA).

### 4.6. Cytochrome P450 (CYP) Enzyme Activities

Two volumes 0.1 M sodium phosphate buffer (pH 7,4) were added to liver tissue samples from SMPD1^+/+^ and SMPD1^+/−^ littermates. The samples were homogenized and subsequently centrifuged for 20 min at 9000× *g* and 4 °C. For determination of the protein content of the resulting 9000× *g* supernatants, a modified Biuret method was used [61]. The following model reactions were performed in the 9000× *g* supernatants to measure CYP enzyme activities: ethoxycoumarin-*O*-deethylation (ECOD) [62]; ethoxyresorufin-*O*-deethylation (EROD) [63]; pentoxyresorufin-*O*-depentylation (PROD) [63]; ethylmorphine-*N*-demethylation (EMND) [64].

### 4.7. Statistics

Mann-Whitney U test (MWU-test) was performed to determine statistically significant differences between groups. A level of *p* ≤ 0.05 was considered statistically significant. With respect to analyses of CYP expression profiles, log2 fold changes of the values of at least ±1 were considered to be of biological significance.
